# Fabrication of a Ternary Nanocomposite g-C_3_N_4_/Cu@CdS with Superior Charge Separation for Removal of Organic Pollutants and Bacterial Disinfection from Wastewater under Sunlight Illumination

**DOI:** 10.3390/toxics10110657

**Published:** 2022-10-29

**Authors:** Malik Imran Afzal, Sammia Shahid, Sana Mansoor, Mohsin Javed, Shahid Iqbal, Othman Hakami, El Sayed Yousef, Foziah F. Al-Fawzan, Eslam B. Elkaeed, Rami Adel Pashameah, Eman Alzahrani, Abd-ElAziem Farouk

**Affiliations:** 1 Department of Chemistry, School of Science, University of Management and Technology, Lahore 54770, Pakistan; 2 Department of Chemistry, School of Natural Sciences (SNS), National University of Sciences and Technology (NUST), H-12, Islamabad 46000, Pakistan; 3 Chemistry Department, Faculty of Science, Jazan University, Jazan, Saudi Arabia; 4 Research Center for Advanced Materials Science (RCAMS), King Khalid University, P.O. Box 9004, Abha 61413, Saudi Arabia; 5 Physics Department, Faculty of Science, King Khalid University, P.O. Box 9004, Abha 61413, Saudi Arabia; 6 Department of Chemistry, College of Science, Princess Nourah bint Abdulrahman University, P.O. Box 84428, Riyadh 11671, Saudi Arabia; 7 Department of Pharmaceutical Sciences, College of Pharmacy, AlMaarefa University, Riyadh 13713, Saudi Arabia; 8 Department of Chemistry, Faculty of Applied Science, Umm Al-Qura University, Makkah 24230, Saudi Arabia; 9 Department of Chemistry, College of Science, Taif University, P.O. Box 11099, Taif 21944, Saudi Arabia; 10 Department of Biotechnology, College of Science, Taif University, P.O. Box 11099, Taif 21944, Saudi Arabia

**Keywords:** ternary nanocomposite, antibacterial activity, catalytic degradation, methylene blue, ciprofloxacin

## Abstract

The synthesis of a photo-catalyst with a narrow bandgap and efficient capability to degrade contaminants in the presence of sunlight is currently challenging but exciting. In this work, an efficient photocatalytic ternary nanocomposite g-C_3_N_4_/Cu@CdS has been synthesized successfully by using the co-precipitation method. The synthesized composite was then characterized by SEM, XRD studies, EDX analysis, and ultra-violet-visible (UV-VIS) spectroscopy. The catalytic efficiency for the methylene blue (MB) dye and drug degradation (ciprofloxacin) was assessed by UV-visible absorption spectra. Gram-positive and Gram-negative bacteria were used to test the fabrication composite’s antibacterial properties. Various compositions (1%, 3%, 5%, 7%, and 9%) of/Cu@CdS nanocomposite (NCs) and 20%, 30%, 40%, 50%, and 60% of g-C_3_N_4_ NCs were prepared. Results reveal that 5%Cu@CdS and 40%g-C_3_N_4_5%Cu@CdS showed maximum antibacterial activity and photocatalytic degradation of dye and drug. The X-ray pattern showed no remarkable change in doped and pristine CdS nanoparticles (NPs). The efficient photocatalytic degradation activity of the fabricated ternary nanocomposite against MB dye and ciprofloxacin an antibiotic drug makes it a viable contender for solving environmental problems.

## 1. Introduction

In recent years, semiconductor photo-catalysis has attained much attention because of its ability to use light energy to drive catalytic reactions. The application of photo-catalyst technology is needed to compete with the environmental pollution and energy shortage problems [1,2,3,4]. TiO_2_ is considered to be the first and most investigated semiconductor, first studied by Fujishima. The drawback was that the TiO_2_ semiconductor works efficiently only with UV (ultraviolet) radiation and thus cannot be used in solar-driven applications. For increasing the efficiency of TiO_2_ semiconductors, TiO_2_ was doped with various metallic and non-metallic elements. The resulting dopants, therefore, do not enhance the photocatalytic activity to a much greater extent [5,6,7]. The development of a photo-catalyst that is stable, abundant, and performs an efficient catalytic activity with visible light is still a challenge of great interest for researchers.

Nowadays, due to exponential population growth, the availability of fresh drinking water has become one of the most concerning worldwide problems [8]. Moreover, drastic industrial development has resulted in the release of untreated waterborne contaminants, including natural organic matter, antibiotics, heavy metal complexes, and microorganisms [9]. Over 7 × 10^5^ to 1 × 10^6^ tons and 100,000 types of various dyes are produced globally each year for use in industries such as leather and textile fabrics, paper printing, synthetic polymers, paints, and pigments [10]. About 10 to 15% of total dyes produced are discharged as waste, either into industrial effluents or the surrounding environment. These pollutants eventually reach freshwater sources, which cause various health problems including allergy, dermatitis, cancer, dysfunction of kidneys, and hormonal and reproductive malfunctions in humans [11]. Over the past few decades, researchers have made strenuous efforts to mitigate the deterioration of this indispensable natural resource around the world. Advanced photocatalytic oxidation processes employing semiconductors, such as CdS [12], TiO_2_ [13,14,15,16], and ZnO [17], have attracted the attention of experts because of their remarkable effectiveness in eliminating water impurities by photo-degradation while producing no carcinogenic waste [18].

Graphitic carbon nitride(g-C_3_N_4_), a polymeric compound comprised of carbon, nitrogen, graphene and a minor amount of hydrogen content, has been introduced as a metal-free photo-catalyst that exhibits solar-driven applications [19] and water purification applications [20,21]. The use of this organic semiconductor is advantageous because of its economic feasibility, ease of availability, relatively high stability, and most important intrinsic visible light response [22,23,24,25]. Tian et al. reported different dimensional structures of g-C_3_N_4_ photo-catalyst. For example, 0D quantum dots, 1D nanorods, nanotubes, 2D nanosheets, and 3D nano-spheres and nano-flowers were designed to investigate photocatalytic efficiency [26]. Liao et al. investigated the g-C_3_N_4_-based composite photo-catalysts for HER application [27]. The photocatalytic response can be enhanced by various methods such as the formation of porous structures, using metal or non-metal elements as doping materials, and coupling with graphene [28]. In this work, graphene-coupled semiconducting material was fabricated and its photocatalytic efficiency was examined. Creating a stable photo-catalyst with a high optical absorption capacity and that exhibits the ability to improve the separation of photo-generated charges is still challenging [29]. 

In the past few years, various semiconductor NPSs (CdS, CdSe, ZnSe, CuO, ZnO) were prepared by using different fabrication techniques [30,31,32,33,34,35]. Among all of the above semiconductor nanoparticles, CdS and NPs are of great importance because of their distinctive physical and chemical properties [36]. Cadmium sulfide (CdS) exhibits a wide band gap, with a band gap energy of 2.42 eV [37]. CdS nanoparticles show high photocatalytic efficiency for the wastewater treatment and catalytic degradation of dyes (MB). The heterojunction semiconductor g-C_3_N_4_/Cu@CdS is used as a photocatalyst to investigate the efficiency of fabricated nanocomposites for dye and drug degradation. Bacterial infections are one of the most serious health problems that humans face. The growth of infectious diseases caused by pathogenic strains, the emergence of bacterial antibiotic resistance, and the development of new bacterial mutations have piqued the interest of researchers who are looking for new ways to combat these organisms. Another concern connected with bacteria is biofilm formation, which can lead to serious medical and industrial issues [38].

Herein, we have fabricated a novel cadmium sulfide (CdS)-based heterojunction photo catalyst composite by co-precipitation and provided a panorama of its photocatalytic mechanism, photocatalytic and optical properties under different morphologies. To the best of our knowledge, a ternary composite with this composition has not been reported before. Additionally, we have especially emphasized the relationship between the doping concentrations and photo and antibacterial activity. The percentage composition of the g-C_3_N_4_ substrate in the final composite was also given special attention regarding its synthesis and properties. SEM, XRD, EDX, and UV-visible spectroscopy were used to analyze the produced nanocomposite. The novel catalyst series synthesized in this study may be potentially applied for the treatment of textiles and other industrial wastewater sources which discharge toxic organic dyes and antibiotic drugs in the effluents.

## 2. Experimental Work

### 2.1. Chemicals

Without additional purification, analytical-grade compounds of all types were utilized. In deionized water, the solutions were made. (Cd(Ac)_2_.2H_2_O, 98%) and copper sulfate (CuSO_4_.5H_2_O) were obtained from Sigma Aldrich. Urea (NH_2_)_2_CO, and thiourea (NH_2_)_2_CS) were obtained from AppliChem, Germany while sodium hydroxide (NaOH) was acquired from Omicron, China.

### 2.2. Preparation of the Photo-Catalyst

#### 2.2.1. Synthesis of Graphitic Carbon Nitride g-C_3_N_4_

g-C_3_N_4_ was synthesized by calcination of urea. About 8–9 g of urea was heated in a muffle furnace at about 5 °C per minute temperature rise until 550 °C, and kept at this temperature for 2 h [39]. The crucible was left in the furnace for cooling to ambient temperature. An off-white fluffy solid was obtained which was pulverized into a fine powder and preserved for further use.

#### 2.2.2. Synthesis of Cadmium Sulfide Nanoparticles 

The co-precipitation process, which was modified somewhat from that used by Xu et al., was employed to create CdS NPs [40]. Cadmium acetate dehydrates and thiourea was used as precursors for Cd and S, respectively. A total of 25 mL of 0.25 M solutions of CdAc_2_ were stirred for 20 min with a 0.2 g Polyvinylpyrrolidone (PVP) capping agent for 30 min at 80 °C. pH was adjusted at 11–12 using a 4 M NaOH solution. A total of 30 mL of 0.25 M thiourea solution was added dropwise while stirring strongly at 80°C. Yellow-colored CdS precipitates were produced, and they were filtered before being repeatedly washed with deionized water, absolute ethanol, and ethanol. Precipitates were then dried at 80 °C for 2 h. The solid obtained was pulverized to a fine powder. 

#### 2.2.3. Synthesis of Copper-Doped Cadmium Sulfide

Chemical doping of CdS was done by adding dopants before the precipitates were formed. A total of 0.05 M CuSO_4_ solution was used for doping. The method given in Section 2.2.2 was repeated by adding 2.5, 7.5, 12.5, 17.5 and 22.5 mL of 0.05 M CuSO_4_ solution after PVP addition for preparing 1, 3, 5, 7 and 9% of Cu-doped photo-catalyst.

#### 2.2.4. Synthesis of Ternary g-C_3_N_4_/Cu@Cds Nanocomposite Photo-Catalyst

**Figure 1:**
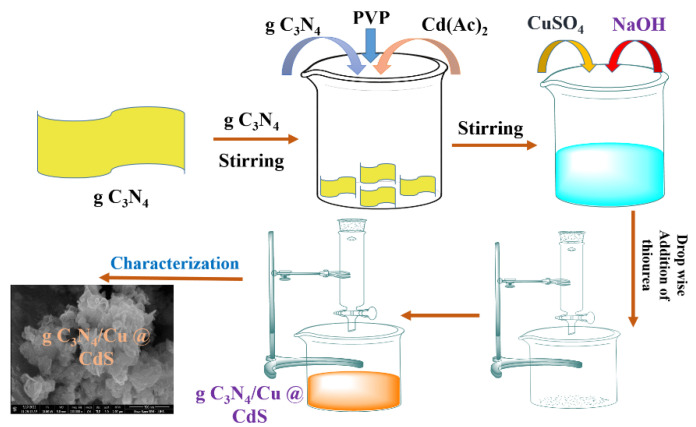
Schematic illustration of synthesis of g-C_3_N_4_/Cu@Cds nanocomposite.

g-C_3_N_4_, already prepared in 2.2.1, was used in this section. Heterogeneous ternary composites were synthesized by adding 0.144, 0.246, 0.383, 0.575, 0. 8625, and 1.342 g of g-C_3_N_4_ to obtain the composite with 20, 30, 40, 50, 60, and 70% of g-C_3_N_4_, respectively. To create a fine suspension, these quantities were dispersed individually in deionized water and agitated for an hour. Method 2.2.3 was followed subsequently. A total of 25 mL of 0.25 M solutions of CdAc_2_ were added to this suspension with a 0.2 g Polyvinylpyrrolidone (PVP) capping agent. A total of 12.5 mL of 0.05 M CuSO_4_ solution was added, followed by stirring for 30 min at 80 °C. pH was adjusted at 11–12 using a 4 M NaOH solution. 30 mL of 0.25 M thiourea solution was added dropwise. Yellow-colored precipitates of CdS were obtained which were filtered and washed. The formation of g-C_3_N_4_/Cu@Cds nanocomposite is shown in (Figure 1). 

## 3. Characterization

Crystallinity and the crystalline phase composition were analyzed by XRD using an X-ray diffractometer (XRD, Bruker D2-Phaser, Dublin, Ireland). The morphology of the fabricated samples was determined by scanning electron microscopy (SEM) and elemental proportions by energy-dispersive spectroscopy (EDS) using FEI Nova 450 NanoSEM. The UV-visible spectrophotometer was used to monitor and record dye degradation, which was used to gauge the photocatalytic performance of the catalysts (Cary 60 UV-Vis, Agilent, Santa Clara, CA, USA).

## 4. Photo-Degradation Activity

### 4.1. Degradation of Methylene Blue

To study the efficiency of the catalysts, photodegradation of the model pollutant methylene blue (MB) was studied with and without a catalyst. CdS NPs and Cu-doped (1%, 3%, 5%, 7% and 9%) CdS NPs were individually applied as described in a later section. Among the composites, (20, 30, 40, 50, 60, 70%) g-C_3_N_4_ with 5%Cu@CdS NPs were evaluated for their degradation activity. To order to achieve adsorption/desorption equilibrium before exposing to the light, 80 mL of MB solution (20 mg/L) containing 50 mg of each catalyst was mixed vigorously for 20 min in the dark. Every 20 min, 5 mL of the sample was taken, centrifuged for 10 min to remove the catalyst, and then a UV-visible spectrum from 200 nm to 800 nm was acquired.

### 4.2. Degradation of Ciprofloxacin

Ciprofloxacin (CIP) was used as a model drug to study the photocatalytic degradation of drug pollutants with and without catalysts. CdS NPs, Cu-doped (1%, 3%, 5%, 7% and 9%) CdS NPs and composites (20, 30, 40, 50, 60, 70%) g-C_3_N_4_-5%Cu@CdS NPs) were evaluated for their photocatalytic activity. A total of 80 mL of CIP solution (25 mgL^−1^) was mixed with 30 mg of each of the catalysts and stirred in darkness for 20 min to reach adsorption/desorption equilibrium between the pollutant and the catalyst. The mixture was then transferred to Petri dishes before exposure to solar radiation [41]. Every 15 min, 5 mL of each sample was taken, centrifuged for 10 min to remove the catalyst, and then a UV-visible spectrum from 200 nm to 800 nm was acquired.

## 5. Antibacterial Activity 

The antibacterial activity of the photo-catalysts (CdS, Cu@CdS NPs, g-C_3_N_4_, and g-C_3_N_4_-Cu@CdS composite) for *Staphylococcus aureus* (*S. aureus*) and *Escherichia coli* (*E. coli*) bacteria was carried out using the agar well diffusion approach. The agar well diffusion method was used to test the antibacterial activity of the photo-catalysts (CdS, Cu@CdS NPs, g-C_3_N_4_, and g-C_3_N_4_-Cu@CdS composite) for Gram-positive and Gram-negative bacteria [42]. *Staphylococcus aureus* (Gram-positive) and *Escherichia coli* were the bacterial strains employed to test the antibacterial capabilities (Gram-negative). The bacterial strains came from the PCSIR laboratory facility in Lahore, Pakistan. For the positive control, ciprofloxacin was utilized as a typical antibiotic, while distilled water served as the negative control.

## 6. Results and Discussion

### 6.1. Scanning Electron Microscope (SEM) Analysis

**Figure 2:**
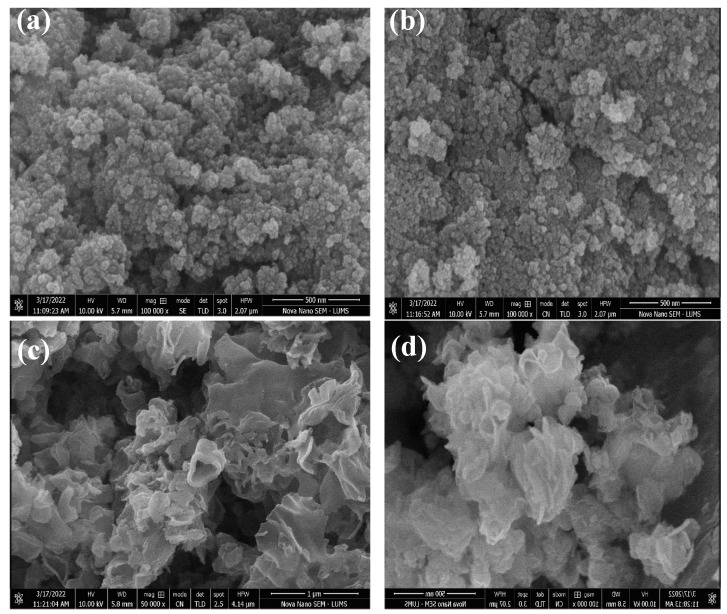
SEM analysis of (**a**) CdS NPs (**b**) Cu@Cds (**c**) gC_3_N_4_ (**d**) g-C_3_N_4_/Cu@CdS.

(Figure 2a–d) represents the SEM images of CdS NPs, Cu@Cds, gC_3_N_4,_ and g-C_3_N_4_/Cu@Cds nanocomposite. The nanocomposite was synthesized by the co-precipitation method. The morphology and size of the fabricated nanocomposite were investigated by SEM (Nova Nano SEM-LUMS). The CdS NPs possess a spherical shape and nanoparticles were agglomerated. The surface of Cu-doped CdS NPs appears to be smooth and has a small grain-like shape with irregular growth that may be due to Ostwald ripening. The graphitic carbon nitride g-C_3_N_4_ exhibits a flaky texture and the g-C_3_N_4_-Cu@Cds nanocomposite have a flaky texture.

### 6.2. EDX Spectroscopy

**Figure 3:**
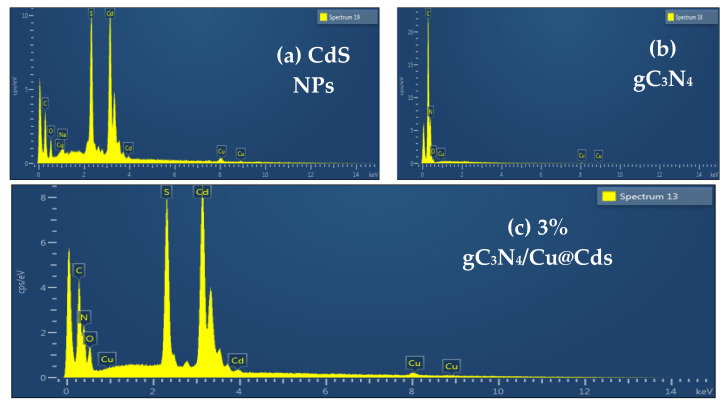
EDX pattern of (**a**) CdS NPs, (**b**) gC_3_N_4_ and (**c**) 3% g-C_3_N_4_/Cu@Cds nanocomposite.

EDX is an analytical technique that provides information about the elemental composition of synthesized materials. When a material is hit by electromagnetic radiation, it emits X-rays, which were then analyzed by using the EDX technique. The sample is targeted by a high-energy laser in an EDX instrument. (Figure 3a–c) shows the EDX spectra of gC_3_N_4_ CdS NPs, and 40%g-C_3_N_4_-5%Cu@Cds nanocomposite. In (Figure 3a) the appearance of cadmium (Cd) and sulfur (S) peaks show the presence of these elements in CuS nanoparticles. (Figure 3b) indicates the presence of carbon (C) and nitrogen (N) present in graphitic carbon nitride. (Figure 3c) displays the EDX spectra of/g C_3_N_4_/Cu@Cds nanocomposite. 

### 6.3. XRD and XPS Analyses

**Figure 4:**
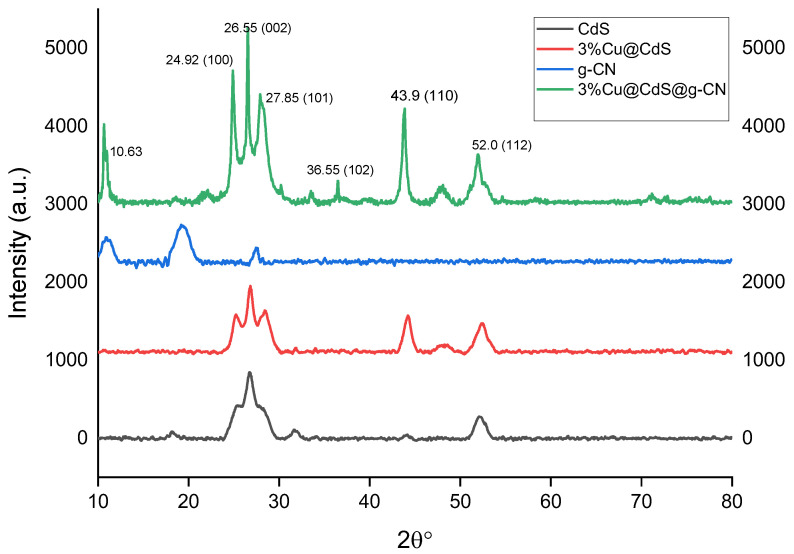
XRD pattern of CdS NPs, gC_3_N_4,_ and 5% g-C_3_N_4_/Cu@Cds nanocomposite.

All materials’ phases and crystalline structures were determined using XRD analysis. The virgin and Cu-doped CdS nanoparticles corresponded with the wurtzite structure, according to the XRD patterns in Figure 4 (cod ref code 96-900-8863) [43]. The almost complete absence of peaks demonstrated that the 5%Cu@CdS nanocomposite structures were well matched with the hexagonal phase. According to an analysis of the XRD peaks, the peaks at 2θ = 24.92°, 26.55°, 27.85°, 36.55°, 43.90°, and 52.05° were produced, respectively, by the contributions of the (100), (002), (101), (102), (110) and (112) reflection planes. The CdS photo-diffraction catalyst’s peaks at 2 = 24.860, 26.540, 28.240, 43.890, 47.940, and 51.910 were attributed to contributions from the reflection planes (100), (002), (101), (110), (103), and (112), respectively. The XRD peaks associated with the (100), (002), (101), and (112) reflection planes of CdS were identified in the ZnO/CdS photocatalyst at 2θ = 25.17°, 26.65°, 28.23°, and 52.23°, respectively. Additionally, the effective integration of the Cu dopant was shown by two diffraction peaks at 2θ = 44.280 and 48.130. The peak intensity of the doped CdS phase is noticeably stronger as compared to CdS alone, as a few peaks in pristine CdS are missing. The peak 2θ = 10.63 in the final composite was detected due to impurity. As seen in Figures S1 and S2, 5% g-C_3_N_4_/Cu@Cd nanocomposite formation and the electronic states of each of its component parts were determined using XPS analysis. The XPS examination further confirmed the results of the SEM and EDX that the 5% g-C_3_N_4_/Cu@CdS included g-C_3_N_4_, CdS, and Cu.

### 6.4. Degradation 

#### 6.4.1. Photocatalytic Degradation of Dye (MB)

**Figure 5:**
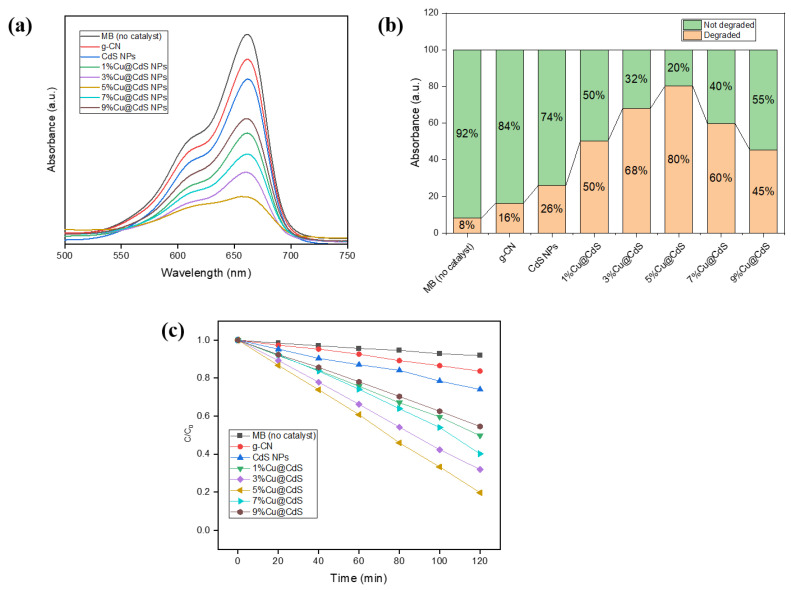
UV-visible spectra of photocatalytic degradation of methylene blue with g-C_3_N_4_, CdS NPs, Cu-doped CdS NPs: (**a**) absorption curves, (**b**) percentage dye degradation, (**c**) relative degradation curves C/C_0_.

Figure 5a–c shows how the synthetic Cu-doped CdS nanoparticles combined with g-C3N4 degraded the standard pollutant methylene blue (MB) under sunshine, respectively. The highest absorption was seen at 662 nm in all spectral graphs. The intensity of blue dye was decreased initially by adsorption on the catalyst’s surface and further decreased by photo-degradation [44,45]. A decrease in the intensity of peaks showed that MB was oxidized to secondary compounds when exposed to sunlight in presence of a synthesized catalyst. All the samples were exposed to the sun for 120 min, measured to be an average 90–100 kLux using a Lux light meter, during March and April at the University of Management and Technology, Lahore (31.4835° N, 74.4121° E), Punjab, Pakistan.

**Figure 6:**
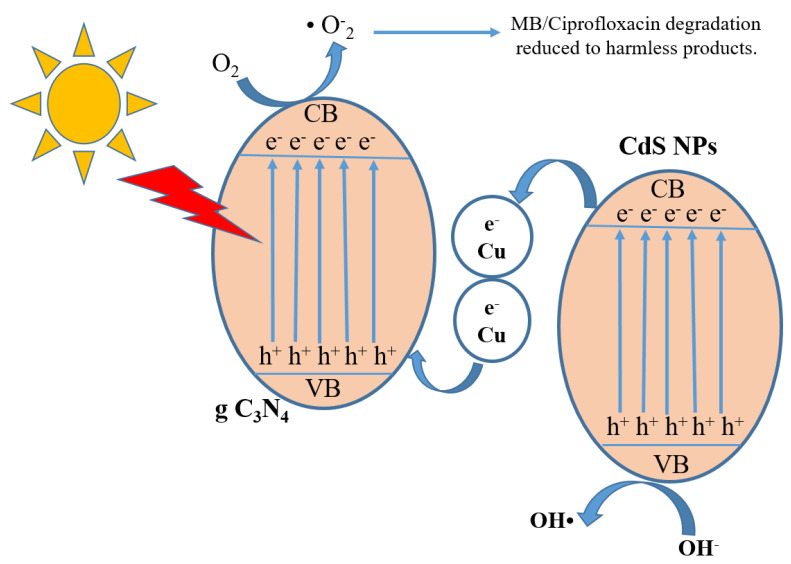
Mechanism of photocatalytic activity.

Figure 5a shows that 5%Cu@CdS nanoparticles proved to be the most efficient catalyst, which decomposed a net 80% of the dye after adsorption. Furthermore, efficiency was increased by fixing the doped nanoparticles on g-C_3_N_4_ to fabricate the composite (Figures S3 and S4). The composite 40%g-C_3_N_4_/5%Cu@CdS showed 95% MB degradation. This increase in efficiency is because of a decrease in band gap by adding Cu and making it a Z-scheme catalyst by merging with g-C_3_N_4_. The mechanism of photocatalytic degradation is shown in (Figure 6).

#### 6.4.2. Photocatalytic Degradation of the Drug (CIP)

**Figure 7:**
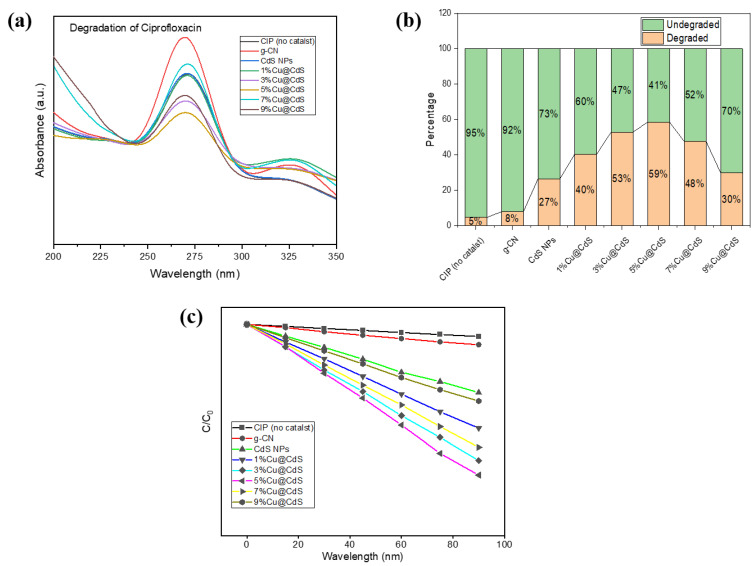
UV-visible spectra of photocatalytic degradation of Ciprofloxacin with g-C_3_N_4_, CdS NPs, Cu-doped CdS NPs: (**a**) absorption curves, (**b**) percentage dye degradation, (**c**) relative degradation curves C/C_0_.

All the samples were exposed to solar radiation for 90 min, measured to be an average of 80 k Lux using the Lux light meter, during the months of February–March at the University of Management and Technology, Lahore (31.4835° N, 74.4121° E), Punjab, Pakistan. (Figure 7a–c) illustrates the degradation of CIP by the synthesized Cu-doped CdS nanoparticles and their composites with g-C_3_N_4_, respectively. Maximum absorption was shown at λ_max_ = 270 nm. The concentration of CIP decreased by incident light and 5%Cu@CdS NPs proved to be most efficient among Cu-doped CdS NPs series (59% in 90 min) and 40%g-CN/5%Cu@CdS among composites (76% in 90 min). A decrease in the intensity of peaks showed that CIP successfully degraded secondary compounds when exposed to sunlight in presence of a synthesized catalyst.

### 6.5. Scavenging Activity

**Figure 8:**
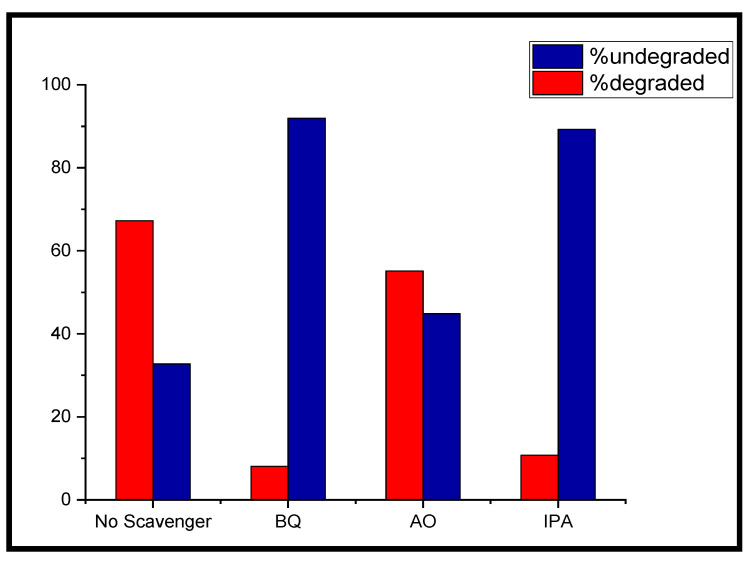
Influence of different scavengers on the photo-degradation of MB with 40%g-C_3_N_4_/5%Cu@CdS.

For studying the photo-degradation mechanism of MB by the g-C_3_N_4_/Cu@CdS catalyst, the role of the species ^•^OH, H^+^, and, ^•^O^2−^ responsible for the degradation of MB in the photo-degradation reaction were examined, as given in (Figure 8). To establish the role of the leading active species, benzoquinone (BQ), isopropanol (IPA), and ammonium oxalate (AO) was added to remove superoxide (^•^O^2−^), the hydroxyl radical (^•^OH) and holes (h^+^), respectively. With the addition of AO to the MB dye solution, the degradation activity of the 40%g-C_3_N_4_/3%Cu@CdS catalyst was reduced to 82% (to 76.8% without scavenger), whereas the addition of IPA and BQ showed comparatively little reduction (12% and 16%, respectively) in degradation of MB under the same conditions of light, temperature and pH of dye solution. This confirmed that the generation of holes (h^+^) plays a key role in the degradation of MB. The photo-generated electrons and superoxide radicals are not as significant as photo-generated holes [46]. 

Two mechanisms—the “type II-heterojunction” and the “straight Z-scheme”—were proposed in light of the aforementioned findings (Figure 8). The presence of copper supported by g-C_3_N_4_ has enhanced the charge separation increasing the electron-hole recombination time. The differences in the standard potential of conduction bands of CdS, Cu, and g-C_3_N_4_ enable the photo-generated electrons to be accommodated in Cu^2+^ ions.

### 6.6. Antibacterial Activity

**Figure 9:**
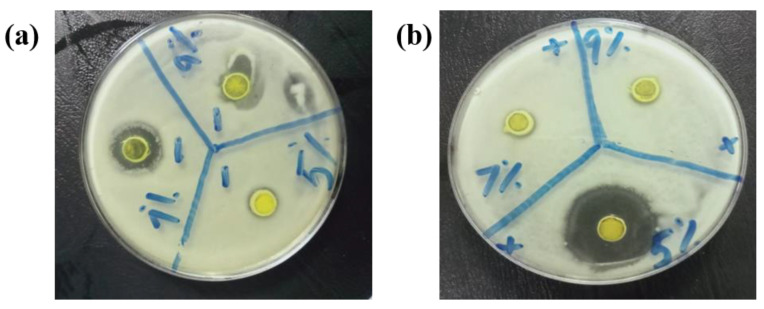
(**a**). The antimicrobial activity of; a. 7%Cu@CdS against S. aureus (**b**). 5%Cu@CdS against *E. coli*.

Stock suspensions of each of CdS, Cu@CdS NPs, g-C_3_N_4_, g-C_3_N_4_/CdS, and g-CN/Cu@CdS NCs were prepared in distilled water by ultra-sonication to yield a final concentration of 50 mg mL^−1^, 100 mg mL^−1^, 150 mg mL^−1^, and 200 mg mL^−1^. Suspensions were stored at room temperature and sonicated once again for 20 min just before adding to the assay. The reference antibiotic Ciprofloxacin concentration was 1 mg mL^−1^. The antibacterial activities were studied using agar well diffusion method. Muller–Hinton agar plates were prepared for testing antibacterial activity. The prepared inoculum of *S. aureus* (Gram-positive) and *E. coli* (Gram-negative) bacteria was spread on plates. Wells were made using sterilized steel boring and filled using micropipette with 20 µL of CdS nps in a concentration of 200 mg/mL, 150 mg/mL 100 mg/mL and 50 mg/mL each. The method was repeated using Cu@CdS NPs, g-C_3_N_4_, g-C_3_N_4_/CdS, and g-CN/Cu@CdS NCs. Each MHA plate was incubated at 37 °C for 24 h. After incubation, the zone of inhibition was measured as shown in Figure 9. 

The 5%Cu@CdS showed the greatest antibacterial activity against *S. aureus* and 7%Cu@CdS was found to be most effective against *E. coli bacteria*. g-CN/Cu@CdS NCs showed a weaker action against both G-positive and G-negative bacteria.

## 7. Conclusions

In summary, g-C_3_N_4_/Cu@CdS NCs were successfully synthesized by the chemical co-precipitation method which showed photocatalytic properties in the visible region of the light spectrum. The interval of electron-hole pairs recombination of CdS np was remarkably increased when doped with Cu. Serving as a perfect electron carrier, g-C_3_N_4_ gave stability to the doped CdS. The photocatalytic efficiency was maximized, with 95% degradation of MB in 120 min and 75% of CIP in 90 min, with 40% g-C_3_N_4_ contents by weight. The antibacterial activity of synthesized nanocomposite against S. aureus and E. coli was studied. The antibacterial study showed that 40%g-C_3_N_4_-5%Cu@CdS NCs and 40%g-C_3_N_4_-7%Cu@CdS NCs have significant antibacterial activity against Gram-positive and Gram-negative bacteria, respectively. The fabricated ternary nanocomposite 40%gC_3_N_4_/5%Cu@CdS NCs exhibited superior photocatalytic activity over CdS NPs, g-C_3_N_4_, and 5%Cu@CdS nanomaterials against dye degradation of methylene blue (MB). The 40%g-C_3_N_4_/5%Cu@Cds showed 95% degradation of MB as compared to 80% degradation by 5%Cu@Cds and 26% by pristine CdS NPs in 90 min under the sun. Similar efficiency was observed in drug degradation (Ciprofloxacin). 40%g-C_3_N_4_/5%Cu@Cds reduced the drug concentration by 76% whereas 5%Cu@Cds and CdS NPs showed up to 59% and 27% degradation, respectively.

## Data Availability

The data will be available on request.

## Supplementary Materials

The following supporting information can be downloaded at: https://www.mdpi.com/article/10.3390/toxics10110657/s1. Figure S1. High-resolution XPS spectra of 5% g-C3N4/Cu@CdS nanocomposite; (a) Cd 3d, (b) S 2p, (c) Cu 2p and (d) C 1s. Figure S2. High-resolution N 1s XPS spectra of 5% g-C3N4/Cu@CdS nanocomposite. Figure S3. UV-Visible Spectra of Photocatalytic degradation of Methylene Blue with g-C3N4, CdS NPs, Cu doped CdS NPs (a) Absorption curves (b) Percentage dye degradation (c) Relative degradation curves C/C0. Figure S4. UV-Visible Spectra of Photocatalytic degradation of Ciprofloxacin with g-C3N4, CdS NPs, Cu doped CdS NPs (**a**) Absorption curves (b) Percentage dye degradation (c) Relative degradation curves C/C0.
