# Supplementary material for: Fabrication of a Ternary Nanocomposite g-C3N4/Cu@CdS with Superior Charge Separation for Removal of Organic Pollutants and Bacterial Disinfection from Wastewater under Sunlight Illumination
Source: Toxics. 2022 Oct 29;10(11):657. doi: 10.3390/toxics10110657 (PMC9699158; doi:10.3390/toxics10110657)
Supplement: Supplementary file 1 [file toxics-10-00657-s001.zip › toxics-1969406-supplementary.pdf]

Article

Fabrication of a Ternary Nanocomposite g-C_3N_4/Cu@CdS with Superior Charge Separation for Removal of Organic Pollutants and Bacterial Disinfection from Wastewater under Sunlight Illumination

Malik Imran Afzal [1], Sammia Shahid [1], Sana Mansoor [1], Mohsin Javed [1], Shahid Iqbal [2,*], Othman Hakami [3], El Sayed Yousef [4,5], Foziah F. Al-Fawzan [6], Eslam B. Elkaeed [7], Rami Adel Pashameah [8], Eman Alzahrani [9] and Abd-ElAziem Farouk [10]

[1] Department of Chemistry, School of Science, University of Management and Technology, Lahore 54770, Pakistan

[2] Department of Chemistry, School of Natural Sciences (SNS), National University of Sciences and Technology (NUST), H-12, Islamabad 46000, Pakistan

[3] Chemistry Department, Faculty of Science, Jazan University, Jazan, Saudi Arabia

[4] Research Center for Advanced Materials Science (RCAMS), King Khalid University, P.O. Box 9004, Abha 61413, Saudi Arabia

[5] Physics Department, Faculty of Science, King Khalid University, P.O. Box 9004, Abha, Saudi Arabia

[6] Department of Chemistry, College of Science, Princess Nourah bint Abdulrahman University, P.O. Box 84428, Riyadh 11671, Saudi Arabia; ffalfozan@pnu.edu.sa

[7] Department of Pharmaceutical Sciences, College of Pharmacy, AlMaarefa University, Riyadh 13713, Saudi Arabia

[8] Department of Chemistry, Faculty of Applied Science, Umm Al-Qura University, Makkah 24230, Saudi Arabia

[9] Department of Chemistry, College of Science, Taif University, P.O. Box 11099, Taif 21944, Saudi Arabia

[10] Department of Biotechnology College of Science, Taif University, P.O. Box 11099, Taif 21944, Saudi Arabia

* Correspondence: shahiducas@gmail.com

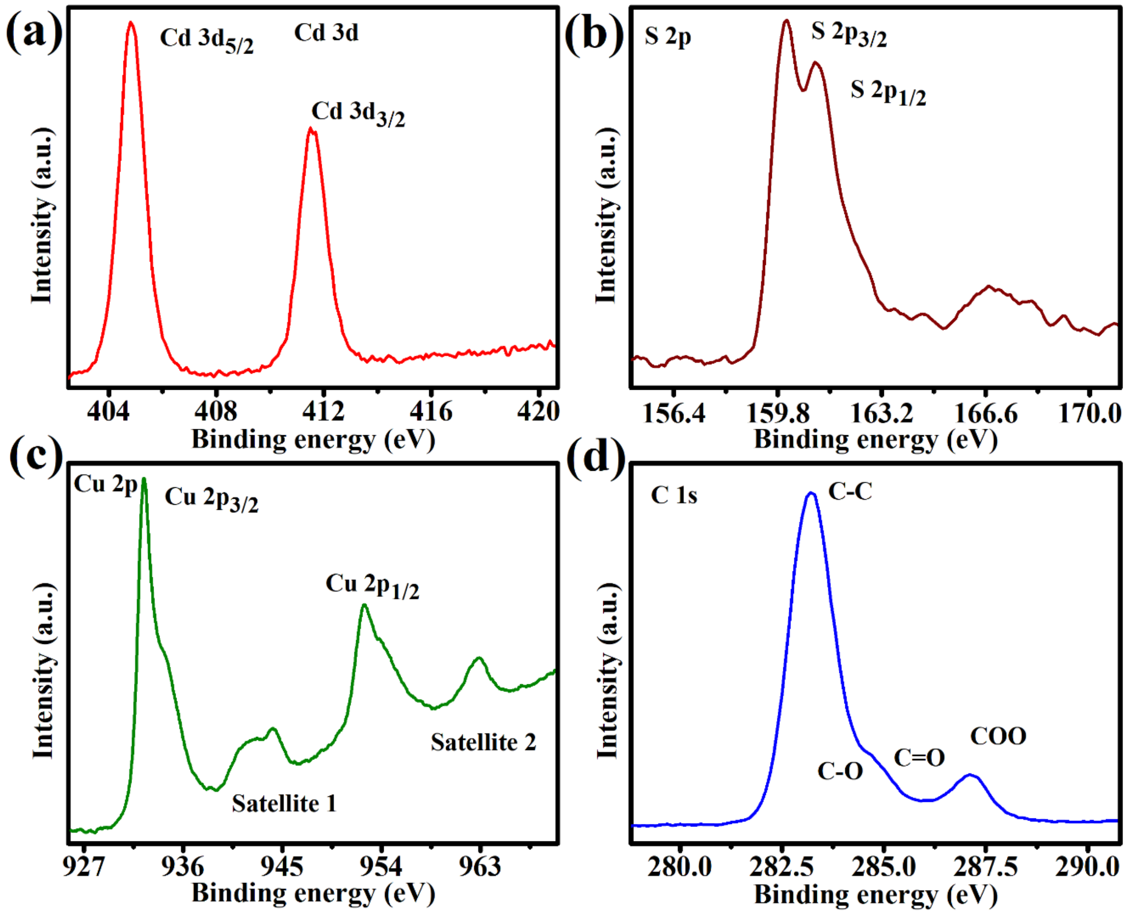

Figure S1. High-resolution XPS spectra of 5% g-C_3N_4/Cu@CdS nanocomposite; (a) Cd 3d, (b) S 2p, (c) Cu 2p and (d) C 1s.

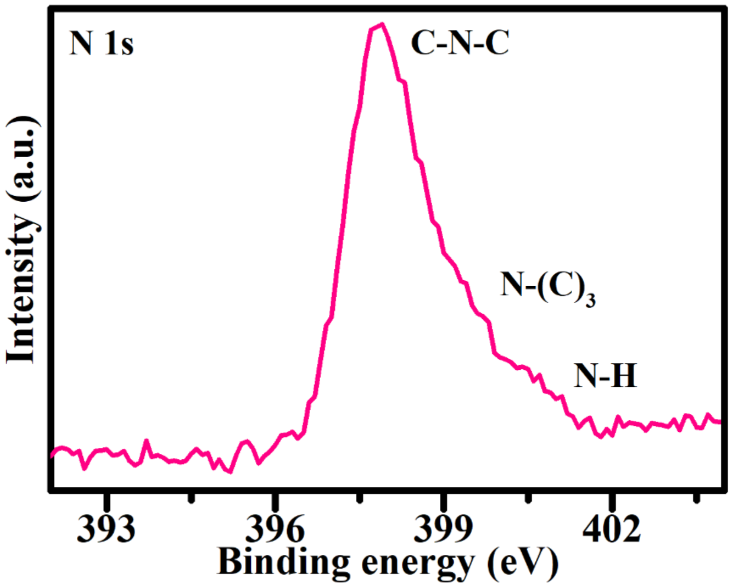

Figure S2. High-resolution N 1s XPS spectra of 5% g-C_3N_4/Cu@CdS nanocomposite.

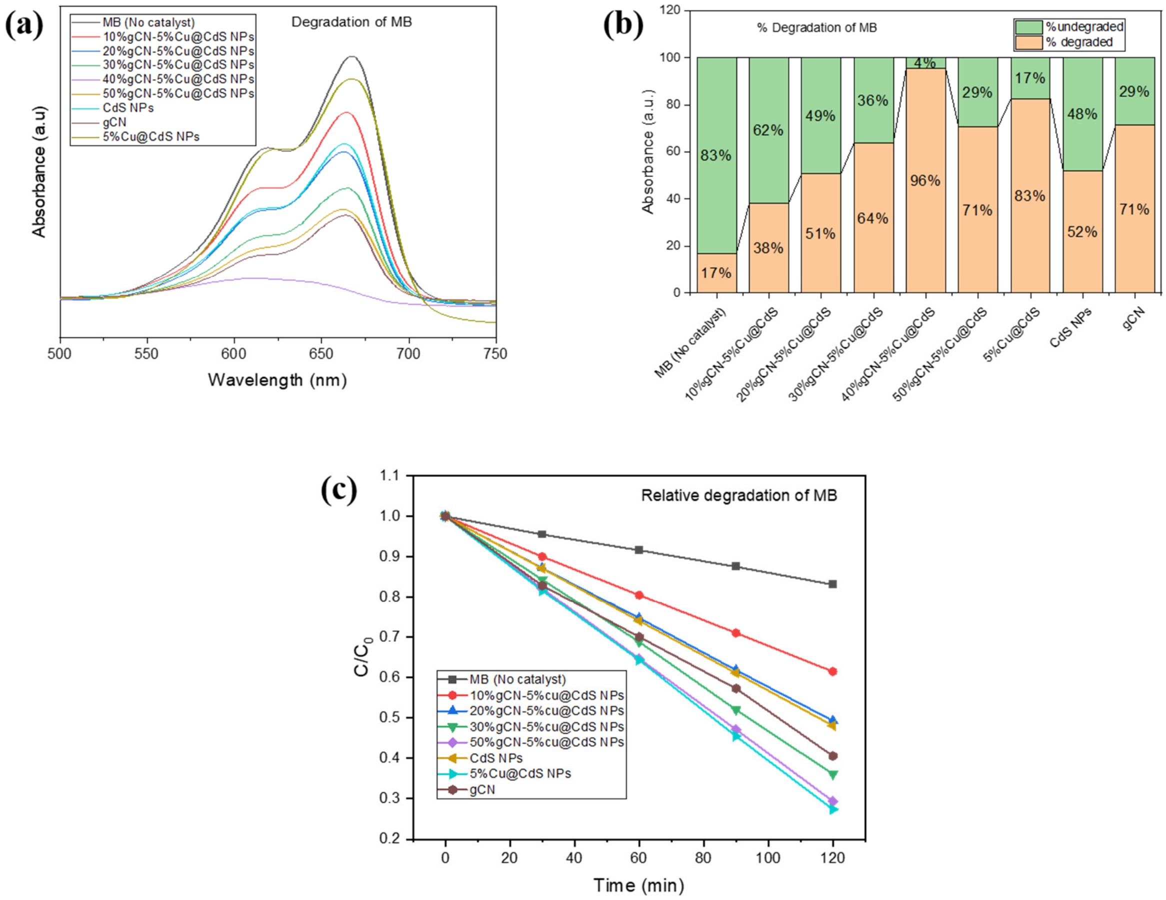

Figure S3. UV-Visible Spectra of Photocatalytic degradation of Methylene Blue with g-C_3N_4, CdS NPs, Cu doped CdS NPs (a) Absorption curves (b) Percentage dye degradation (c) Relative degradation curves C/C_0.

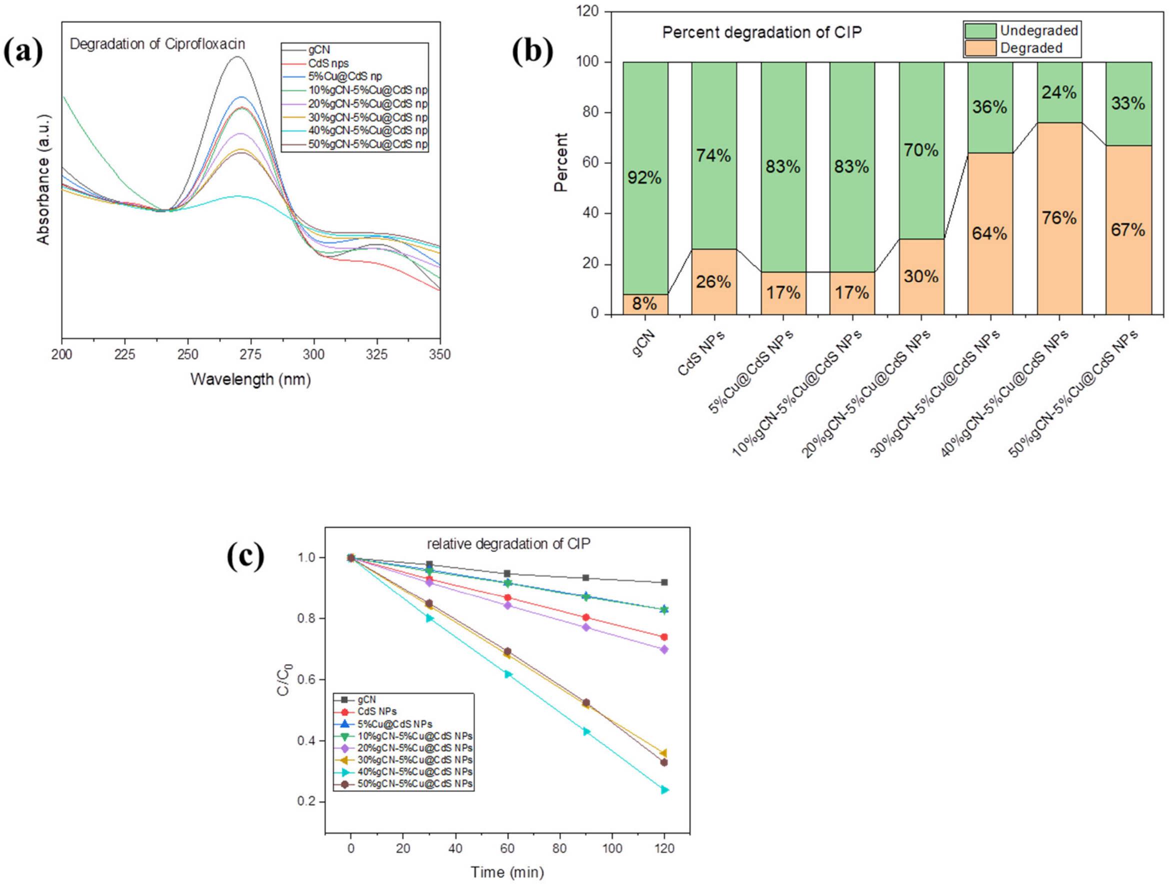

Figure S4. UV-Visible Spectra of Photocatalytic degradation of Ciprofloxacin with g-C_3N_4, CdS NPs, Cu doped CdS NPs (a) Absorption curves (b) Percentage dye degradation (c) Relative degradation curves C/C_0.
